# The Impact of Hypoglycemia on Productivity Loss and Utility in Patients With Type 2 Diabetes Treated With Insulin in Real-world Canadian Practice: Protocol for a Prospective Study

**DOI:** 10.2196/35461

**Published:** 2022-03-28

**Authors:** Veronique Lambert-Obry, Jean-Philippe Lafrance, Michelle Savoie, Jean Lachaine

**Affiliations:** 1 Faculty of Pharmacy Université de Montréal Montreal, QC Canada; 2 Faculty of Medicine Université de Montréal Montreal, QC Canada

**Keywords:** real-world evidence, work productivity, health-related quality of life, diabetes, hypoglycemia

## Abstract

**Background:**

Type 2 diabetes mellitus (T2DM) imposes a substantial burden owing to its increasing prevalence and life-threatening complications. In patients who do not achieve glycemic targets with oral antidiabetic drugs, the initiation of insulin is recommended. However, a serious concern regarding insulin is drug-induced hypoglycemia. Hypoglycemia is known to affect quality of life and the use of health care resources. However, health economics and outcomes research (HEOR) data for economic modelling are limited, particularly regarding utility values and productivity losses.

**Objective:**

This real-world prospective study aims to assess the impact of hypoglycemia on productivity and utility in insulin-treated adults with T2DM from Ontario and Quebec, Canada.

**Methods:**

This noninterventional, multicenter, 3-month prospective study will recruit patients from 4 medical clinics and 2 endocrinology or diabetes clinics. Patients will be identified using appointment lists and enrolled through consecutive sampling during routinely scheduled consultations. To be eligible, patients must be aged ≥18 years, diagnosed with T2DM, and treated with insulin. Utility and productivity will be measured using the EQ-5D-5L questionnaire and Institute for Medical Technology Assessment Productivity Cost Questionnaire, respectively. Questionnaires will be completed 4, 8, and 12 weeks after recruitment. Generalized estimating equation models will be used to investigate productivity losses and utility decrements associated with incident hypoglycemic events while controlling for individual patient characteristics. A total of 500 patients will be enrolled to ensure the precision of HEOR estimates.

**Results:**

This study is designed to fill a gap in the Canadian evidence on the impact of hypoglycemia on HEOR outcomes. More specifically, it will generate productivity and utility inputs for the economic modeling of T2DM.

**Conclusions:**

Insulin therapy is expensive, and hypoglycemia is a significant component of economic evaluation. Robust HEOR data may help health technology assessment agencies in future reimbursement decision-making.

**International Registered Report Identifier (IRRID):**

PRR1-10.2196/35461

## Introduction

### Background

Diabetes imposes a substantial burden because of its increasing prevalence and life-threatening complications. According to Diabetes Canada, 1 in 3 Canadians is living with diabetes or prediabetes, among which type 2 diabetes mellitus (T2DM) accounts for 90% to 95% of cases [1]. Glycemic control is key to diabetes management, and several types of oral therapies have been approved for T2DM. In patients who do not achieve glycemic targets on oral antidiabetic drugs, initiation of insulin therapy is recommended [1]. However, a serious concern regarding insulin is drug-induced hypoglycemia [2]. Hypoglycemic events are a major obstacle to the achievement of glycemic targets and represent a challenge for both patients and physicians. The severity of hypoglycemia is defined by clinical manifestations ranging from mild symptoms to seizure and coma [2].

### Current State of Knowledge

Hypoglycemia is known to affect quality of life and the use of health care resources. However, health economics and outcomes research (HEOR) data for economic modelling are limited, particularly regarding utility values and productivity losses. O’Reilly et al [3] recently published data on health care costs associated with hypoglycemia based on the Canadian cohort of the International Hypoglycemia Assessment Tool study. Although relevant data on direct costs were provided, productivity losses were subject to recall bias (1-year recall) and were limited to absenteeism (excluding presenteeism and unpaid work). Regarding utility values, a highly cited literature review by Beaudet et al [4] identified utilities for 20 modeling complications associated with T2DM. For hypoglycemia, the review found only 1 study with utility estimates suitable for economic modeling, that is, considering the number of events (rather than the presence vs absence of hypoglycemia). Economic modeling of T2DM is particularly challenging because multiple interrelated organ systems are involved over a long period, with numerous risk factors. For an adverse event such as hypoglycemia, HEOR data should depend on the number of episodes to fit T2DM economic models, as in the study by Currie et al [5], the current preferred source for utility values for hypoglycemia. Although the study by Currie et al [5] is the source recommended by several health technology assessment (HTA) agencies, it is criticized by the National Institute for Health and Care Excellence (NICE) for being subject to selection bias and recall bias [6,7]. It should be noted that Beaudet et al [4] also highlighted that variability around the utility estimates (eg, SE) is not always reported, thereby limiting future sensitivity analyses in cost-utility analyses (CUAs).

Canadian data on productivity losses due to hypoglycemia include a 2005 survey of 133 patients with T2DM of whom up to 9% and 26% missed work or studies following nonsevere hypoglycemic events (NSHEs) and severe hypoglycemic events (SHEs), respectively [8]. A web-based survey included 150 patients with T2DM having a nocturnal NSHE in the previous month. Among the 87 working respondents, 15 (17%) reported an average of 3.5 hours of lost work [9]. In the Canadian Hypoglycemia Assessment Tool cohort, 6% (8/134), 3.8% (5/134), and 7.5% (10/134) of T2DM workers reported on average 2.9 days taken off (SD 21.2), 3.8 days arriving late (SD 8.8), and 1.7 days leaving early (SD 0.7), respectively, based on a 1-year recall [3]. As for utility, time trade-off (TTO) values were elicited from 51 Canadian respondents with diabetes and 79 respondents from the general population. The mean utility ranged between 0.85 and 0.94, 0.77 and 0.90, and 0.66 and 0.83 for rare hypoglycemic events (quarterly), intermediate (monthly), and frequent (weekly) health states, respectively. In a multivariate linear regression, the estimated utilities associated with a single hypoglycemic event (all types combined) were −0.0033 and −0.0032 for respondents with diabetes and the general population, respectively [10]. Another TTO study estimated utility decrements of 0.006, 0.008, 0.059, and 0.062 for daytime NSHEs, nocturnal NSHEs, daytime SHEs, and nocturnal NSHEs, respectively [11]. A third TTO study including patients with diabetes and respondents from the general population estimated utility decrements ranging from 0.0028 to 0.0056, 0.0076, 0.0592 to 0.0726, and 0.0616 to 0.0826 for daytime NSHEs, nocturnal NSHEs, daytime SHEs, and nocturnal SHEs, respectively [12]. No Canadian studies that met the Canadian Agency for Drugs and Technologies in Health (CADTH) recommendation to use an indirect method for utility measurement (eg, EQ-5D) were identified.

### Study Rationale and Relevance

The population at risk of drug-induced hypoglycemia is significant, with nearly 20% to 30% of patients with T2DM requiring insulin [13,14]. In the last 5 years, CADTH has reviewed 9 economic evaluations in T2DM, among which 3 were CUAs for insulin therapies [15-17]. Considering the growing interest in HEOR data in insulin-treated patients with T2DM, there is a need for robust estimates. Data on productivity losses are lacking, which are particularly meaningful in Quebec, where the *Institut national d’excellence en santé et en services sociaux* preconizes economic evaluations by adopting a societal perspective. In addition, in 3 recent pharmacoeconomic reports on insulin therapies for T2DM, CADTH highlighted the uncertainty around utility values for hypoglycemia [15-17]. The committee described the current evidence on the impact of hypoglycemia on utility as limited and of low quality. In CADTH’s reanalyses for the 3 CUAs, variation in utility values for hypoglycemia led to a significant change in the estimates of quality-adjusted life-years gains. The incremental cost-effectiveness ratios were very sensitive to any changes in utility decrements for hypoglycemic events and were therefore determined to be an important driver of the results [15-17]. Thus, there is a need for HEOR data that are robust enough to make an informed decision with reasonable uncertainty. The aim of this real-world prospective study is to assess the impact of hypoglycemia on productivity and utility in insulin-treated adults with T2DM from Ontario and Quebec, Canada.

## Methods

### Research Purpose and Study Design

This noninterventional, multicenter, 3-month prospective study is designed to collect HEOR inputs for future economic modeling. This study will generate descriptive HEOR estimates that can be incorporated into T2DM models, along with precision parameters (ie, CIs and SEs) to evaluate uncertainty in CUAs in sensitivity analyses. Considering the nature of the disease, a longitudinal design with repeated measures was deemed appropriate to limit confounding and increase the quality of evidence.

### Study Population

In Canadian clinical practice, patients with T2DM who are on insulin can be followed up by a family physician or a specialist [18]. Therefore, patients will be recruited from 4 medical clinics and 2 endocrinology or diabetes clinics. To increase generalizability, patients will be recruited from the 2 largest Canadian provinces, Ontario and Quebec. Patients will be identified using appointment lists and enrolled through consecutive sampling during routinely scheduled consultations. To be eligible, patients must be aged ≥18 years, diagnosed with T2DM, and treated with insulin. There are no restrictions on insulin regimens (eg, short-acting, long-acting, or mixed insulin). Given the lack of reimbursement, the use of insulin pumps is limited in the treatment of T2DM [19]. Patients must also be able to understand and read English or French and provide informed consent. Patients will be excluded if they participate in a clinical trial. A screening log will be used to document the participation rates.

### Definition of Hypoglycemia

According to the Canadian Diabetes Association and the American Diabetes Association (ADA), hypoglycemia is defined as plasma glucose concentrations of ≤3.9 mmol/L (≤70 mg/dL) [2,20]. To reflect a real-world setting, the definition of hypoglycemia in this study is not restricted to documented episodes but includes any hypoglycemic event as judged by the patient. Symptoms defining hypoglycemia include trembling, palpitations, sweating, anxiety, hunger, nausea, tingling, difficulty concentrating, confusion, weakness, drowsiness, impaired vision, difficulty speaking, headache, and dizziness. Patients will self-report hypoglycemic episodes recorded by either symptoms or blood glucose testing alone or a combination of both. This approach has also been used in several large real-world studies [18,19,21-24] and better represents real-life practice where patients do not always test their blood glucose level for different reasons (eg, forgetting, neglecting, lack of knowledge, or lack of testing materials). Hypoglycemic events will be categorized as either severe or nonsevere. An SHE is defined as an event requiring assistance from another person (to administer carbohydrates, to administer glucagon, or take other corrective actions), whereas an NSHE can be managed by the patient alone, as per the Canadian Diabetes Association and ADA definitions [2,20].

Along with the severity of hypoglycemic events, the frequency of events promotes the fear of future hypoglycemia [2,20]. Fear itself affects patients’ quality of life [2,20]. In addition to the physical symptoms due to hypoglycemic events, the negative emotional impact of the fear of hypoglycemia is also a concern. For modeling purposes, HTA agencies recommend that the assessment of the impact of hypoglycemia on utility should consider both the severity and frequency of episodes. They also recommend not applying a utility decrement separately for the fear of hypoglycemia. Therefore, this study was designed to capture the overall impact of hypoglycemia on utility, including the transient effects of events and the resulting fear.

### Measurement of Outcomes

#### Utility

The EQ-5D-5L questionnaire will be used to measure the utility values [25,26]. This validated instrument is widely used and covers five dimensions (mobility, self-care, usual activities, pain or discomfort, and anxiety or depression) that are further divided into five levels (no problems, slight problems, moderate problems, severe problems, and extreme problems). The EQ-5D (EQ-5D) health states may be converted into a single summary index through a crosswalk value set. An EQ-5D summary index of 1 represents complete health, 0 represents death, and negative values represent states worse than death. The questionnaire also includes a visual analog scale that records the respondent’s self-rated health using a vertical scale ranging from 0 to 100 with end points labeled as “the best health you can imagine” and “the worst health you can imagine.” EQ-5D scores represent patients’ health status on the day of questionnaire completion.

#### Productivity Loss

The Institute for Medical Technology Assessment (iMTA) of Erasmus University Rotterdam recently developed and validated a questionnaire to estimate productivity losses, referred to as the iMTA Productivity Cost Questionnaire (iPCQ) [27]. The *i*PCQ is a generic questionnaire designed to determine the value of productivity losses for economic evaluations adopting a societal perspective. The questionnaire included three modules: lost productivity at paid work because of absenteeism, lost productivity at paid work because of presenteeism (reduced productivity), and lost productivity at unpaid work. A recall period of 4 weeks is used for presenteeism and unpaid work. Absenteeism is divided into two categories: short-term absence (no longer than 4 weeks) and long-term absence (sick leave that started before the recall period).

### Study Procedures

#### Data Collection

The sociodemographic and clinical characteristics of the patients will be collected at recruitment. Patients will complete a baseline questionnaire to report their age, gender, ethnicity, education, income level, employment status, living status, alcohol consumption, smoking, and physical activity. As a proxy for frailty, the self-reported number of visits to a health care provider over the last 3 months and the number of emergency visits and hospitalizations over the last 6 months will also be collected [28]. As a proxy for lifestyle habits, immunization status (pneumococcal, influenza, and COVID-19) will also be self-reported. Self-care (disease management activities) will be assessed using the Diabetes Self-Management Questionnaire, a validated questionnaire covering behaviors related to glycemic control (diet, blood glucose monitoring, medication adherence, physical activity, and contact with health care professionals) [29]. The type of device used for glucose monitoring will also be recorded (eg, traditional finger-prick monitor and flash glucose monitoring device). It should be noted that continuous glucose monitoring systems are not reimbursed in T2DM, thereby limiting their use. To describe the history of hypoglycemia, patients will also be asked if they have experienced NSHEs and SHEs over the last 3 months and the last year, respectively. The duration of diabetes, glycated hemoglobin (HbA_1c_), BMI, therapy, duration of insulin, and vascular complications will be extracted from the patients’ medical records.

#### Assessments and Study Calendar

**Figure 1:**
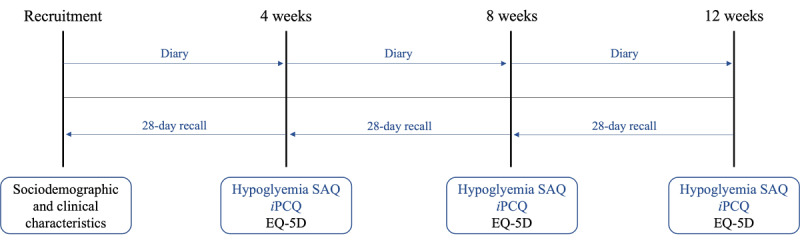
Study calendar. iPCQ: institute for Medical Technology Assessment Productivity Cost Questionnaire; SAQ: self-assessment questionnaire.

Patients will complete a self-assessment questionnaire (SAQ) at 4, 8, and 12 weeks to report the number of NSHEs and SHEs experienced in the last 28 days. Patients will also be provided with a diary to prospectively record hypoglycemic events. Both the SAQ and the diary will be used to estimate the number of hypoglycemic events. The EQ-5D and *i*PCQ will also be completed at 4, 8, and 12 weeks. The study calendar is shown in Figure 1. The questionnaires will be completed electronically via a web-based platform. To ensure that patients complete their questionnaires in a timely manner, reminders will be sent to them via email. Paper-based questionnaires can be provided to participants who do not have access to or are less familiar with using the internet (phone call reminders).

### Ethical Considerations

This study will be conducted in accordance with the Declaration of Helsinki [30]. Approval will be taken from an independent review board before the initiation of the study, and each patient will have to provide written informed consent. After receiving independent review board approval, the protocol will be registered on ClinicalTrials.gov. All study documents, including validated versions of questionnaires, will be made available to participants in English or French, according to their preferences. The patients will not incur any costs for volunteering to participate in this study. Patients will receive a compensation of CAD $20 (US $15.75) for their participation after completion of each questionnaire.

### Statistical Analysis

#### Presentation of Results

Data will be screened for accuracy, and questionnaires completed in paper format (if any) will entail double data entry to minimize transcription errors. Baseline characteristics of all patients participating in the study will be presented. Categorical variables will be summarized as frequencies (number and proportion), and descriptive statistics (mean and SD) will be provided for continuous variables. Hypoglycemia will be measured as hypoglycemic events and treated as a continuous variable, as recommended by HTA agencies for economic modeling of T2DM [6,7]. If hypoglycemic events are reported at a higher rate in the patient’s diary than in the SAQ, diary values will be used to calculate the number of events, and SAQ values will be used in the sensitivity analysis. For the EQ-5D-5L, questionnaire scores will be calculated according to the EuroQoL scoring manual [31] using the Canadian crosswalk value set [32]. For the *i*PCQ, productivity losses will be calculated for each module (absenteeism, presenteeism, and unpaid work) using the iMTA scoring manual [33] and summed up to a total number.

#### Independent Impact of Hypoglycemia

Generalized estimating equation (GEE) models will be used to investigate productivity losses and utility decrements associated with incident hypoglycemic events while controlling for individual patient characteristics. For utility, two separate models will be presented: one for the EQ-5D index and one for the visual analogue scale. For productivity losses, one main analysis will be presented for the total number, and separate models for each module will be presented as a complementary analysis. The potential model-confounding covariates are presented in Table S1 in Multimedia Appendix 1. Independent variables were identified based on existing literature and previous work from the Alliance for Canadian Health Outcomes Research in Diabetes [34]. Diabetes complications (micro- and macrovascular) to be included in the models are those preconized by HTA agencies for CUAs in T2DM [4,6,7]. On the basis of the current reference study for hypoglycemic utility values [5], the number of hypoglycemic episodes will be log-transformed to facilitate model fitting. The original scale will be tested using sensitivity analyses. Moreover, transformations of continuous independent variables will also be tested in the sensitivity analyses (Table S1 in Multimedia Appendix 1). The presence of multicollinearity will be tested, where important collinearity will be defined as a variance inflation factor of >10. Owing to the short duration of the study, all covariates, except hypoglycemia, will be considered time invariant for model simplicity. If vascular complications occur during the study period (incident cases), a history of macrovascular and microvascular complications will be treated as a time-varying covariate. The main models will not include interaction terms, as large-scale studies evaluating the impact of diabetes complications on utility have not demonstrated any interaction between vascular events and event history and among the different vascular events [35-37]. A sensitivity analysis will be performed to test for the interaction between hypoglycemic events and determinants, namely, a history of hypoglycemia and diabetes complications.

Distributions of outcomes will be examined considering the expected nature of productivity data (right-skewed and excess zeros) and utility data (left-skewed, censored, and ceiling effect). Although utility is commonly left-skewed, the distribution of utility scores may vary greatly between health conditions depending on the severity. Left skewness is more common when the studied condition is associated with mild symptoms, whereas severe health states may even lead to negative values representing conditions worse than death [38]. One major advantage of the GEE is the avoidance of distributional assumptions, where several distributions can be tested. Several large-scale longitudinal studies evaluating the impact of diabetes complications on utility, including the landmark United Kingdom Prospective Diabetes Study, used models fitted under a linear framework [35-37]. Common alternatives to the Gaussian distribution with identity link function include the Gaussian distribution with log link, negative binomial distribution with log link, gamma distribution with identity link, and gamma distribution with log link. The gamma distribution requires nonzero positive continuous data; thus, utility must be transposed into disutility (1−utility value). A beta binomial distribution can also be used with a transformation (linear transformation or rescaling) to fit the restrictive open interval (0,1), which excludes the end points 0 and 1 [39]. For the use of health care resources, nonlinear options mostly include negative binomial distribution (for resource use) and gamma and inverse-Gaussian distribution with log link function (for costs). Raw outcome data will be explored, where distributions will be depicted using histograms and normal probability plots. The selection of the model will be based on predictive performance and goodness of fit, with the lowest values for the root mean square error and the mean absolute error and the highest values for pseudo-*R*^2^. Moreover, the model performance will also be calculated using the quasi-likelihood under the independence model criterion (QIC), where the lowest values are the best. QIC and QIC*u* will be used for correlation structure selection and variable selection, respectively. As no distributional assumptions about the response variable are made, the regression parameters cannot be estimated using the maximum likelihood method. Thus, quasi-likelihood statistics will be used (pseudo-*R*^2^, QIC, and QIC*u*) instead of the well-known likelihood statistics (*R*^2^, Akaike information criterion, and Bayesian information criterion) [40]. Different variance-covariance structures are available to fit the relationship between responses. In health economics, common choices include exchangeable correlation structure (observations from the same cluster are equally correlated), autoregressive (correlation decreases with time), and unstructured (different and complex correlations). According to the Good Research Practices for Retrospective Database Analysis Task Force recommendations by the International Society for Pharmacoeconomics and Outcomes Research, if results are similar with different matrices, an exchangeable matrix will be used [41]. Regression diagnostics will be performed to explore the presence of influential observations and outliers (Cook distance and residual plots). Exclusion of extreme values will be tested in sensitivity analysis. All results will be expressed as coefficients, SEs, 95% CIs, and associated *P* values (2-sided tests at the .05 significance level). Full regression models and adjusted effects will be presented. All statistical analyses will be performed using R (R Foundation for Statistical Computing) [42].

#### Sample Size

As per good research practices, the sample sizes for utility estimates should be based on precision rather than hypothesis testing [38,43]. Indeed, there is no consensus on the use of minimally important differences in EQ-5D measures for statistical power calculations [31,44]. Therefore, the sample size was determined to ensure reasonable variability around the utility value for SHEs (which also ensures precision for NSHEs as the incidence is significantly higher). However, no value for the expected SD was found in the literature for performing a reliable precision-based sample size calculation. A literature review was performed to identify studies that estimated utility values for hypoglycemia. However, it is often difficult to find an expected SD for utility values because they are sensitive to the type of instrument, country, precise definition of outcome, or timeframe. Furthermore, most studies did not provide uncertainty values around the estimates. As discussed by Beaudet et al [4], utility values for hypoglycemia are limited and CIs are scarce.

Therefore, the required sample size was determined based on the study by Currie et al [5], which is used as a reference for hypoglycemic utility values by CADTH and NICE [6,7]. Currie et al [5] obtained utility estimates from 68 patients who experienced at least one SHE over a 3-month period. According to Canadian real-world studies, the 3-month incidence of SHEs among insulin-treated patients with T2DM was approximately 17% [18,19,45]. With a 3-month incidence rate of 17%, 400 patients will have to be recruited to ensure that 68 patients experience at least one SHE during the study follow-up. To account for attrition, the sample size will be increased by 20% for a target number of 500 enrolled patients. It is assumed that this sample size will also ensure the precision of the productivity estimates.

#### Missing Data

Missing data will be defined according to outcome-specific guidelines, and a descriptive analysis will be conducted. According to the EQ-5D guidelines, situations that should be treated as missing data include not only unit nonresponse (no completion of questionnaire) but also item nonresponse (incomplete questionnaire) and ambiguous values (eg, 2 boxes are ticked for a single dimension). When completing the *i*PCQ, some questions can be skipped if they are not applicable (eg, when the patient is not employed). If the questions necessary to score a module are incomplete, the module is defined as missing.

If ≤5% of the data are missing and there is no significant difference between completers and noncompleters, missing data will be assumed to be missing completely at random (MCAR). Under the MCAR assumption, the available case analysis performed with a GEE model yields valid estimates [40,46-48]. If >5% of the data are missing, the missing data pattern will be categorized as monotonic (ie, no return after dropout) or nonmonotonic (ie, intermittent missing data). Moreover, the mechanism by which data are missing will be investigated by examining which baseline covariates and previous measurements predict the missingness of a given outcome. On the basis of the results of the regression analysis, a specific variable will be determined to be a predictor of missingness based on statistical significance and clinical plausibility [49]. In the presence of predictors of missingness, data will be considered as not MCAR, which may bias the GEE estimates. This would lead to the use of the weighted generalized estimating equation (WGEE), a recently published extension of the GEE that incorporates an inverse probability weight matrix [50]. WGEE is a valid approach for dropout missingness (monotonic missing pattern), which is the typically observed pattern in longitudinal HEOR studies [51]. In addition, HEOR outcomes are most commonly missing as unit nonresponses (no completion of questionnaire) rather than item nonresponses (incomplete questionnaire) [49,51]. Therefore, the overall questionnaire scores (eg, EQ-5D index score) will be used for missing data analysis. If the use of the WGEE is required, a sensitivity analysis will be conducted to compare the WGEE results with the GEE estimates for the available case analysis.

#### Unmeasured Confounding

The presence of an unknown, unmeasured confounder will be explored using *E* values, which is a new standardized approach for sensitivity analysis [52,53]. For effect estimates, the *E* value is the minimum strength of association on the risk ratio scale that an unmeasured confounder would need to have with both the exposure and outcome, above and beyond the measured covariates, to fully explain the observed association of exposure with the outcome. For the 95% CI limit, the *E* value denotes the minimum strength of association on the risk ratio scale that an unmeasured confounder would need to have with both the exposure and the outcome, above and beyond the measured covariates, to shift the CI to include the null value. This sensitivity analysis will assess the robustness of the associations to unmeasured confounding.

## Results

This study is designed to fill a gap in Canadian evidence on the impact of hypoglycemia on HEOR outcomes. More specifically, it will generate productivity and utility inputs for economic modeling of T2DM. Insulin therapy is expensive, and hypoglycemia is a significant component of economic evaluation. Robust HEOR data may help HTA agencies in future reimbursement decision-making.

## Discussion

### Strengths and Limitations

Insulin-induced hypoglycemia is a burden to patients with diabetes, and this study will collect HEOR estimates reflecting how SHEs and NSHEs negatively affect patients’ productivity and utility. To our knowledge, this would be the first Canadian real-world study to attempt to longitudinally measure the impact of hypoglycemia on utility and productivity loss, including absenteeism, presenteeism, and unpaid work, in insulin-treated patients with T2DM. In this study, hypoglycemia will be categorized into SHEs and NSHEs without further subgrouping by severity (eg, mild or moderate) or time of occurrence (daytime or nocturnal). This classification is preconized by HTA agencies for CUAs in T2DM [6,7]. Subjective measurement of hypoglycemia may overestimate the number of episodes, which may underestimate the outcome values by event (conservative approach). In addition, the recently published guidelines by the International Hypoglycaemia Study Group recommend that the hypoglycemia threshold be lowered to <3.0 mmol/L (<54 mg/dL) in clinical trials [54]. This threshold was suggested because it is sufficiently low to indicate serious, clinically important hypoglycemia and because this level does not occur under physiological conditions in individuals who do not have diabetes. A joint position statement by the ADA and the European Association for the Study of Diabetes was subsequently published, which indicated that the glucose level should be lowered for clinical trials [54]. The use of the official definition of hypoglycemia (≤3.9 mmol/L) instead of a lower threshold might lead to overreporting of nonclinically serious events. Nevertheless, this approach is also conservative as it underestimates the outcome values by event. Defining hypoglycemia based on symptoms or blood glucose measurements is considered a reliable method that best reflects real-world practice [18,19]. The frequency of assessments (recall period) was based on previous Canadian studies [18,19]. The use of diary records (if higher than the number reported in the questionnaire) can compensate for potential recall bias and is a conservative approach as it would decrease the outcome values by event. Considering that longitudinal studies usually have a minimum of 3 measurements and that the frequency of assessments is 4 weeks, the duration of the study is therefore 3 months.

The outcomes will be measured using validated questionnaires. Although several instruments are available to estimate productivity losses, there is no gold standard [55]. In addition, HTA agencies make different recommendations regarding types of productivity losses (absenteeism, presenteeism, and unpaid work) to include in economic evaluations and methods for valuation or monetization (human capital approach and friction approach) [33]. Therefore, productivity losses will be presented as total and type-specific raw scores (number of hours per day) and will not be transposed into monetary value. This approach provides flexibility and allows future economic evaluations to be fit for specific purposes. The *i*PCQ scoring manual presents different valuation methods depending on various scenarios (eg, presence of long-term absences and frequency of measurements) and is fully adaptable to different HTA requirements [33]. It is recommended that the choice of a work productivity instrument for economic evaluations should be based on purpose, perspective, practicality, population, and psychometrics (5 *P*s) [55]. The *i*PCQ meets the 5 *P*s criteria as it is a validated generic instrument that allows monetization, captures all types of productivity losses, and minimizes the burden to patients (has short completion time and is easy to understand). Owing to its recent development, the use of the *i*PCQ is less extended than older questionnaires, which comes with the advantage that questions are optimized based on 3 previously validated instruments [33]. Moreover, this promising instrument has a recall period of 4 weeks, which matches measurements of hypoglycemia while limiting recall bias. Although there is no gold standard, the Work Productivity and Activity Impairment Questionnaire is the most widely used instrument to assess productivity losses [56]. Similar to the *i*PCQ, the Work Productivity and Activity Impairment Questionnaire covers absenteeism, presenteeism, and unpaid work, but over a shorter period. As the recall period is only 7 days, it may not capture all productivity losses due to hypoglycemia if events occur outside of the recall period. Regarding utility, the use of generic preference-based instruments is recommended, among which the EQ-5D is the most extensively used [57]. The EQ-5D asks patients how they feel on that day without any recall period. Therefore, for complications associated with transient effects only (eg, diarrhea), utility should be measured simultaneously (ie, on the same day). When acute events are followed by persistent fear and anxiety (eg, stroke), different measurement timings can be used. If the utility is measured shortly after the event, the punctual effect of the complication will be captured. If utility is not measured concurrently, then a general effect will be captured. For economic modeling of T2DM, utility values recommended by HTA agencies were all sourced from studies designed to capture the general impact of diabetic complications, including hypoglycemia [6,7]. In a reference study by Currie et al [5], the EQ-5D captured decrements in utility due to hypoglycemic events that occurred over the last 3 months. Furthermore, in several randomized controlled trials, the impact of SHEs on utility was measured for up to 1 year after the event [36,58,59]. A limitation of this approach is the underestimation of acute physical effects related to the event. Indeed, the current design may capture the psychological effects of hypoglycemia more accurately. Nevertheless, this study will estimate the overall impact of this treatment-related adverse effect on utility, providing relevant inputs for T2DM economic modeling.

Confounding is a concern in observational research [28]. Before implementing this study, independent variables were thoroughly identified, and each known variable will be measured to limit confounding. To reflect real-world practice, independent variables that will be extracted from patients’ medical files will represent the last available value, which may not reflect the current unknown value. Moreover, some independent variables will be self-reported, potentially leading to residual confounding. Nevertheless, there are no unmeasured known confounders, and the potential impact of an unmeasured unknown confounder will be tested using *E* values [52,53]. There is evidence that a cross-sectional design may overestimate the impact of T2DM complications on utility because of the underlying heterogeneity across patients [37]. Therefore, a longitudinal design with time-varying exposure will help protect against time-invariant confounding (natural heterogeneity) [40,46-48]. A GEE model will be used to account for the correlation associated with repeated measures from the same individual. Although mixed models provide a flexible framework compared with the GEE model, they require a large sample size and may be computationally demanding. Therefore, the simpler GEE method will be used to deal with this noncomplex data set (no large-scale data analysis, single level of clustering, and absence of nonstochastic time-varying covariates, eg, time from baseline). In addition, it is acknowledged that a GEE model is comparable with a random intercept model for continuous outcomes. A drawback of the GEE model is the assumption that the data are MCAR [40,46-48]. Therefore, if the data are not MCAR, the WGEE will be used to ensure the robustness of the estimates [50]. Furthermore, reminders and incentives should help to minimize the rate of missing data.

Patients will be recruited from several regions throughout Quebec and Ontario, Canada, to increase the external validity of the results. However, recruitment sites will be limited to urban areas and may not be representative of rural areas. The real-world design and broad eligibility criteria will ensure that the HEOR estimates are generalizable to a target population for future reimbursement purposes. It is assumed that recruitment through clinical sites only will not affect the representativeness of the sample as patients with diabetes have regular follow-ups with their health care providers, thereby capturing most eligible patients and not only patients in worse condition. Furthermore, enrolling patients from both medical and diabetes clinics will be representative of the target population. Although probability sampling is the gold standard for ensuring sample representativeness, it is often not feasible in Canada because many jurisdictions lack electronic patient databases, particularly family practice. Yet, systematic participant recruitment as consecutive sampling using appointment lists also helps minimize selection bias (including oversampling). Participation rates will be recorded to document the risk of selection bias. It should be noted that the COVID-19 pandemic may affect patient productivity and utility. However, given the use of the GEE model to estimate the independent impact of hypoglycemia on the HEOR estimates, the results are expected to be valid and generalizable.

### Conclusions

Robust evidence on the productivity and utility of insulin-induced hypoglycemia is lacking in Canada. Currently, available data on productivity loss have not been estimated using a validated questionnaire, thereby increasing the risk of bias [3,8,9]. A systematic review published in 2021 identified 42 unique instruments for measuring productivity, and the authors recommended the iPCQ for use in economic evaluations [60]. As for the current evidence on utility decrement due to hypoglycemia, Canadian data are limited to vignette studies (ie, bespoke descriptions of impaired health states), which are not the preferred source of utility owing to their inherent drawbacks [10-12]. This study will generate high-quality HEOR estimates for future economic modeling of T2DM.

## Appendix

Multimedia Appendix 1 Potential independent variables for the generalized estimating equations models.

## Abbreviations

ADA: American Diabetes Association
CADTH: Canadian Agency for Drugs and Technologies in Health
CUA: cost-utility analysis
GEE: generalized estimating equation
HAT: hypoglycemia assessment tool
HEOR: health economics and outcomes research
HTA: health technology assessment
iMTA: institute for Medical Technology Assessment
iPCQ: institute for Medical Technology Assessment Productivity Cost Questionnaire
MCAR: missing completely at random
NICE: National Institute for Health and Care Excellence
NSHE: nonsevere hypoglycemic event
QIC: quasi-likelihood under the independence model criterion
SAQ: self-assessment questionnaire
SHE: severe hypoglycemic event
T2DM: type 2 diabetes mellitus
TTO: time trade-off
WGEE: weighted generalized estimating equation
