# Supplementary material for: The Impact of Hypoglycemia on Productivity Loss and Utility in Patients With Type 2 Diabetes Treated With Insulin in Real-world Canadian Practice: Protocol for a Prospective Study
Source: JMIR Res Protoc. 2022 Mar 28;11(3):e35461. doi: 10.2196/35461 (PMC9002599; doi:10.2196/35461)
Supplement: Multimedia Appendix 1 [file resprot_v11i3e35461_app1.docx]

Supplementary Table 1. Potential independent variables for the generalized estimating equations models

| **Independent variables** | **Reference variables** |
| --- | --- |
| Age | Per additional year^a^ |
| Gender | Dummy: Female |
| Ethnicity | Dummy: White |
| Education | Dummy: Post-secondary |
| Household income | Dummy: ≥$80,000 |
| Employment status | Dummy: Employed |
| Living status | Dummy: Married/Living with partner |
| Alcohol consumption | Dummy: Heavy drinker^b^ |
| Smoking | Dummy: Current smoker |
| Physical activity | Dummy: Inactive^c^ |
| Physician visits | Per additional visit^d^ |
| Emergency visits and hospitalizations | Per additional visit^e^ |
| Self-care | Per additional score unit^f^ |
| Blood glucose monitoring system | Dummy: Flash (continuous) glucose monitoring |
| Influenza immunization | Dummy: Annual influenza vaccination |
| COVID-19 immunization | Dummy: Fully vaccinated^g^ |
| Pneumococcal immunization | Dummy: Vaccinated (ever) |
| Previous experience of NSHE | Dummy: History^h^ |
| Previous experience of SHE | Dummy: History^i^ |
| Duration of diabetes | Per additional year^j^ |
| HbA1c (%) | Per additional unit^k^ |
| Body mass index | Per additional kg/m^2 l^ |
| Combination of therapies | Dummy: Combination of insulin and secretagogues |
| Number of daily injections | Per additional insulin injection^m^ |
| Duration of insulin therapy | Per additional year^n^ |
| Macrovascular complications | Dummy: History^o^ |
| Microvascular complications | Dummy: History^p^ |
| Incident NSHE | Per additional event^q^ |
| Incident SHE | Per additional event^r^ |

NSHE: non-severe hypoglycemic event; SHE: severe hypoglycemic event

1. Sensitivity analysis: will be tested as a dummy variable (≥65 years old).
2. Heavy drinker: having ≥4 drinks in a single day at least once a month in the past year (5 for males).
3. Physical inactivity: <150 minutes/week of moderate and vigorous physical activity.
4. Medical visits: number of visits to primary care doctor and specialists over the last three months.
5. Emergency visits and hospitalizations: number of emergency room visits and hospital admissions over the last six months.
6. Self-care: total score of the Diabetes Self-Management Questionnaire (DSMQ).
7. COVID-19 immunization: Complete vaccination is currently defined as 2 doses.
8. Previous experience of non-severe hypoglycemic events: occurrence over the last three months.
9. Previous experience of severe hypoglycemic events: occurrence over the last year.
10. Sensitivity analysis: will be tested as a dummy variable (≥ 10 years).
11. Sensitivity analysis: will be tested as a dummy variable (≥ 7%).
12. Sensitivity analysis: will be tested as a dummy variable (≥ 30 kg/m^2^).
13. Sensitivity analysis: will be tested as a dummy variable (≥ 5 years).
14. Sensitivity analysis: will be tested as a dummy variable (≥ 3 injections/day).
15. Macrovascular complications: myocardial infarction, ischemic heart disease, heart failure, stroke, peripheral vascular disease. Sensitivity analysis: will be tested per additional complication.
16. Microvascular complications: renal failure, proteinuria, severe vision loss, retinopathy, macular oedema, cataract, amputation, neuropathy, ulcer. Sensitivity analysis: will be tested per additional complication.
17. Non-severe hypoglycemic events: will be log-transformed. Interpretation of log-transformed independent variables is as follows: A 1% increase in *x* changes the dependent variable by coefficient/100. With a 100% increase (equivalent to 1 additional event), the coefficient can be interpreted directly without transformation. Sensitivity analysis: will be tested with original scale.
18. Severe hypoglycemic events: will be log-transformed. Interpretation of log-transformed independent variables is as follows: A 1% increase in *x* changes the dependent variable by coefficient/100. With a 100% increase (equivalent to 1 additional event), the coefficient can be interpreted directly without transformation. Sensitivity analysis: will be tested with original scale.
